# Effectiveness of TKI Inhibitors Combined With PD-1 in Patients With Postoperative Early Recurrence of HCC: A Real-World Study

**DOI:** 10.3389/fonc.2022.833884

**Published:** 2022-03-31

**Authors:** Zixiong Li, Ning Han, Xueying Ren, Yuanjing Zhang, Xiaoyuan Chu

**Affiliations:** ^1^ Department of Oncology, Jinling Hospital, School of Medicine, Nanjing University, Nanjing, China; ^2^ Department of Infection, Changzheng Hospital, Naval Medical University, Shanghai, China

**Keywords:** hepatocellular carcinoma, locoregional therapies, PD-1, recurrence, systemic therapy

## Abstract

**Objective:**

To observe the efficacy of TKI inhibitor combined with PD-1 treatment in patients with early recurrence after radical resection of HCC, and to analyze the factors that affect the efficacy.

**Methods:**

The baseline demographic and clinical data of 58 patients with early recurrence after radical resection of HCC (including surgical resection and liver transplantation) were collected. Recurrence and metastasis were classified into early (< or =2 years) and late phase (>2 years). After systemic drug treatment (sorafenib, lenvatinib, PD-1), the efficacy was evaluated based on the RECIST 1.1 standard. COX regression model was used to analyze the factors affecting PFS and OS in HCC patients. Survival curves were drawn by Kaplan-Meier method.

**Results:**

The study finally included 58 patients who underwent radical resection of HCC, of which 39 were in the TKIs group and 19 were in the TKIs + ICIs combined treatment group. There was no statistical difference in the baseline data of the two groups in HB, PLT, Child-Pugh score and other indicators. Efficacy evaluation results showed that in the 39 TKIs group, 7 patients were PD and 9 patients were PR; while in the 19 TKIs combined with PD-1 group, 2 patients were PD and 6 patients were PR. The median PFS of the TKIs group was 6.2 months, while the median PFS of the TKIs combined PD-1 group was 14.0 months (HR= 0.469, P=0.031). The median OS of the TKIs group was 18.0 months, while the median OS of the TKIs combined with PD-1 group was 35.8 months, an extension of 17.8 months (HR= 0.444, P=0.053).

**Conclusion:**

In the first-line treatment of patients with early recurrence after radical resection of HCC, patients treated with TKIs combined with PD-1 therapy has a survival advantage over those treated with TKIs alone. Ascites, HBV DNA positivity, and high levels of AFP often indicate poor efficacy of systemic drug therapy, suggesting that such patients should be closely monitored after surgery and that comprehensive systemic treatment should be administrated in time to improve the prognosis.

## Introduction

Primary liver cancer has become the 4th common malignant tumor and the 3rd cause of tumor death in China (1). The main pathological type of primary liver cancer is hepatocellular carcinoma (HCC), followed by cholangiocarcinoma and mixed liver cancer. The cancer data for the whole year of 2015 showed that there were 466,000 new cases of HCC and 422,000 deaths due to HCC in China each year, both of which were greater than the total number of other regions in the world (2). The main reason for the high incidence and mortality of HCC is the hepatitis B virus (HBV) pandemic in China.

Although surgical resection is the best treatment for HCC, the postoperative prognosis of patients is not ideal. Recurrence occurs in most patients with early HCC after radical operations such as surgical resection, liver transplantation. At present, HCC patients who relapse after radical surgery are mainly administrated with systemic treatment drugs for advanced liver cancer, including etiological treatment, chemotherapy, targeted drug therapy, checkpoint inhibitors (ICIs) and liver protection therapy. Agents targeting epidermal growth factor receptor (EGFR), mainly including tyrosine kinase inhibitors (TKIs), such as sorafenib or lenvatinib, have been approved as first-line drugs for the treatment of advanced liver cancer (3–6).

The mechanisms of early recurrence and late recurrence after radical resection of HCC are completely different. Patients with early recurrence of HCC (within 2 years) may be related to vascular invasion and residual neoplastci foci (7, 8). Although there are many clinical studies on drugs for advanced HCC at present, systemic drug treatment for postoperative recurrence has not been fully studied. Patients with early HCC were prone to recurrence and metastasis after liver cancer resection, transplantation, and transcatheter arterial chemoembolization (TACE) treatment, due to underlying HBV infection (8–10). The effect of systemic treatment on early recurrence stage HCC patients infected with HBV remains unclear. This study is conducted to throw light on this issue.

## Methods

### Research Object

The subjects of the study were patients with recurrence of intrahepatic tumors after radical operation (including surgical resection, liver transplantation) of HBV-related HCC at Nanjing Jinling Hospital and Shanghai Changzheng Hospital between March 2018 and September 2020. The cohort study design was adopted. Patients involved in this study were regularly followed up, and information of postoperative diagnosis and treatment were collected. The research protocol complies with the ethical code of the 2008 Declaration of Helsinki, and all the research subjects signed an informed consent form.

### Inclusion and Exclusion Criteria

1. The inclusion criteria include: a. Patients pathologically diagnosed with HCC; b. Radical operation of HCC, including patients after resection or liver transplantation; c. Recurrence within 2 years after surgery; d. Age ≥18 years; e. Informed consent.
2. The exclusion criteria include: a. Other pathological types of HCC, including intrahepatic cholangiocarcinoma and mixed liver cancer; b. Patients who have not undergone radical operation of HCC in the past, or have distant metastases; c. Patients with poor general conditions after radical operation, or not suitable for systemic treatment for other reasons; d. Patients who relapse more than 2 years after surgery; e. Patients who refuse to participate in this study.

### Clinical Information

**Figure 1:**
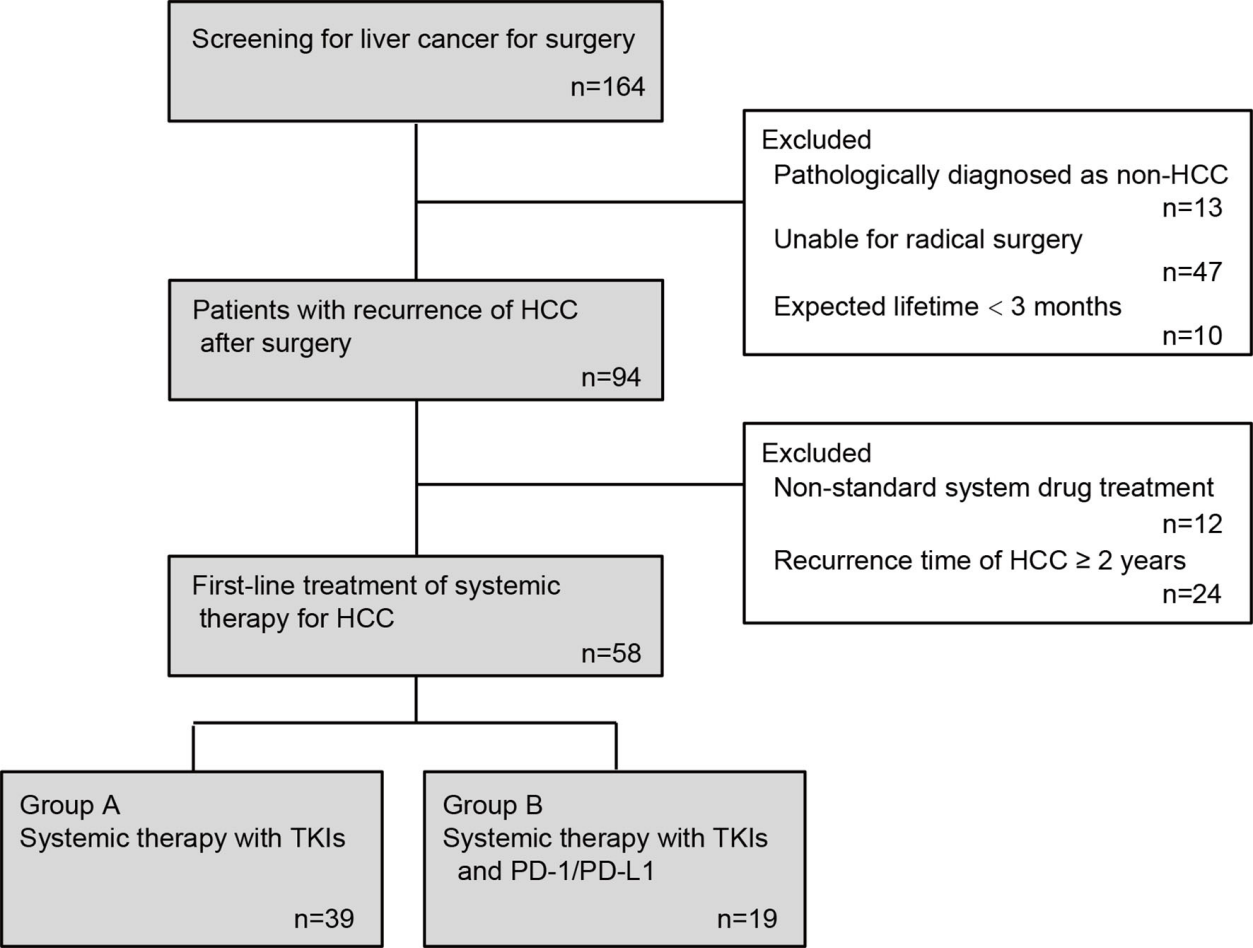
Flow chart of patient enrollment.

After inclusion and exclusion stated above, 58 patients were finally enrolled in the final analysis (**Figure 1**). Baseline sociodemographic information including age, gender, medical record number, contact information, home address, smoking, drinking, and medical history of liver disease and clinical information including laboratory test results within one week before systemic treatment such as liver function indicators, HBV infection indicators (HBsAg, HBsAb, HBeAg, HBeAb and HBcAb), serum HBV DNA levels, and platelet counts were collected from medical records of Nanjing Jinling Hospital and Shanghai Changzheng Hospital.

### Collection of Systemic Drug Treatment Plans and Efficacy Data

For the patients enrolled in this study, in addition to the clinical information after the previous radical operations of HCC in the medical records, information on the subsequent systemic treatment was also collected. The systemic drug treatments involved in this study include 1) oral sorafenib treatment, 400mg, 2/day; 2) oral lenvatinib treatment, body weight ≥60kg, 12mg, 1/day; body weight <60kg, 8mg, 1/day; 3) nivolumab, ivgtt, 3 mg/kg, every 2 weeks; 4) pembrolizumab, ivgtt, 200 mg, every 3 weeks; or 5) toripalimab, ivgtt, 3 mg/kg, every 2 weeks. After the above-mentioned standardized systemic drug treatment, the RECIST 1.1 standard was referred to evaluate the changes in the target lesions in the liver every two months, including disease progression (PD), stable (SD), and partial remission (PR). In addition, follow-up information is fully recorded. All collected patient-related information is accurately entered into computer for subsequent statistical analysis.

### Statistical Methods

Data are all sorted and analyzed using SPSS 18.0 software. COX regression model was conducted to analyze the influence of subgroup factors on PFS and OS in HCC patients, and multivariate analysis was conducted to determine independent risk factors. The survival curve was drawn using the Kaplan-Meier method. The impact of risk factors on the disease outcome was evaluated by HR (hazard ratio). A two-tailed P value less than 0.05 was considered to be statistically significant.

## Results

### Patient Clinical Information

This study finally included 58 patients with HCC recurrence after radical operation, including 39 patients in the TKIs group and 19 patients in the TKIs + ICIs combined treatment group. In the TKIs group, 16 patients were under 50 years old, 19 patients were 50 to 60 years old, and 4 patients were over 60 years old. In the TKIs + ICIs combined treatment group, the above three groups were 5, 4, and 10 cases, which is statistically different from the TKIs group (P=0.002). There were more males in the TKIs group compared with the TKIs + ICIs combined treatment group (89.7% vs 63.2%, P=0.029). There was no statistically significant difference in HB, PLT and other indicators between the two groups. The two groups were comparable at the baseline level (**Table 1**).

**Table 1:** Clinical information of included patients.

Factors	TKIs group, n=39 (%)	TKIs + ICIs group, n=19 (%)	*P* value
age			
<50	16 (41.0)	5 (26.3)	
50-59	19 (48.7)	4 (21.1)	
≥60	4 (10.3)	10 (52.6)	0.002
gender			
female	4 (10.3)	7 (36.8)	
male	35 (89.7)	12 (63.2)	0.029
HB			
≥110g/L	30 (76.9)	13 (68.4)	
<110g/L	9 (23.1)	6 (31.6)	0.488
PLT			
≥100×10^9^/L	22 (56.3)	14 (73.7)	
<100×10^9^/L	17 (43.6)	5 (26.3)	0.203
Child-Pugh			
A	29 (74.4)	11 (57.9)	
B	7 (17.9)	7 (36.8)	
C	3 (7.7)	1 (5.3)	0.286
Tumor stage			
BCLC A	3 (7.7)	4 (21.1)	
BCLC B	33 (56.4)	10 (52.6)	
BCLC C	14 (35.9)	5 (26.3)	0.320
HBV			
negative	4 (10.3)	2 (10.5)	
positive	35 (89.7)	17 (89.5)	0.975
TKIs			
Sorafenib	32 (82.1)	14 (73.7)	
Lenvatinib	7 (17.9)	5 (26.3)	0.460

TKIs, Tyrosine kinase inhibitor; ICIs, Immune checkpoint inhibitor; HB, hemoglobin; PLT, platelet; BCLC, Barcelona Clinic Liver Cancer; HBV, Hepatitis B Virus.

### Effect in Patients With Early Recurrence of Intrahepatic Tumors After Radical Resection of HCC

**Figure 2:**
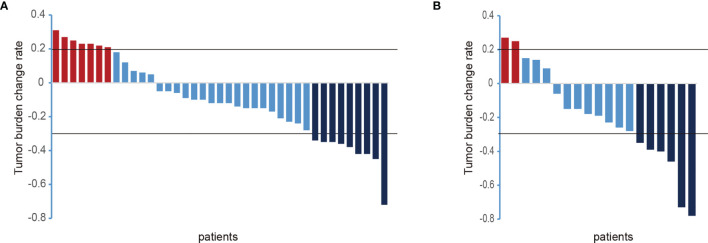
Changes of intrahepatic lesions in the included patients after systemic treatment. [**(A)** TKIs group; **(B)** TKIs + ICIs group; PD, progressive disease; PR, partial response partial remission; ordinate, the sum of the maximum diameter of the tumor target lesion; abscissa, all patients included in this study)].

In the TKIs group, the efficacy evaluation showed that there were 7 patients with PD, 9 patients with PR, and the remaining 23 patients with SD. In the TKIs + ICIs combined treatment group, the efficacy evaluation showed that 2 patients were with PD, 6 patients were with PR, and the remaining 11 patients were with SD. On the whole, after TKIs combined with PD-1 treatment, HCC in the TKIs + ICIs combined treatment is more efficient than TKIs alone (**Figure 2**).

### Survival Analysis

**Figure 3:**
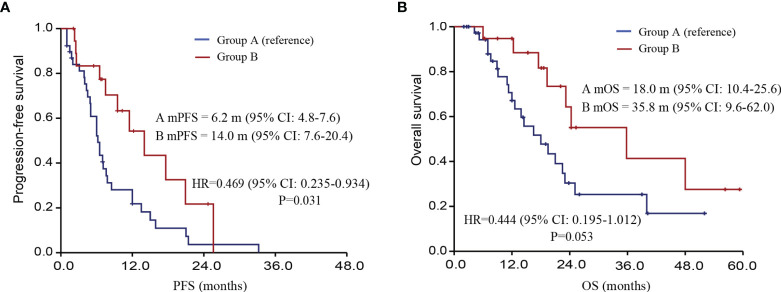
Follow-up information of different treatment options after systemic treatment. [**(A)** PFS of patients; **(B)** OS of patients; PFS, progression-free survival; OS, overall survival].

Univariate COX regression analysis was conducted to determine the effect of treatment group on PFS and OS. The median PFS of the TKIs treatment group and TKIs + ICIs combined treatment group were 6.2 months and 14.0 months (HR=0.469, P=0.031). The median OS of the TKIs treatment group and TKIs + ICIs combined treatment group were 18.0 and 35.8 months, respectively (HR= 0.444, P=0.053) (**Figure 3**).

### Subgroup Analysis

Furthermore, the impact of the above-mentioned baseline biochemical and virological indicators on the OS was analyzed. The results of univariate COX regression analysis showed that patients with ascites had poorer OS than those without ascites, HR (95% CI): 2.611 (1.157-5.892) (P=0.021); patients with positive HBV DNA also had poorer OS than those without, HR (95% CI): 3.451 (1.309-9.102) (P=0.012); patients with high AFP expression, HR (95% CI): 3.631 (1.449-9.097) (P=0.006) (**Table 2**).

**Table 2:** Subgroup analysis of patients with OS.

Factors	Univariate analysis HR (95% CI)	*P* value	multivariate analysis HR (95% CI)	*P* value
gender				
female	1			
male	0.853 (0.363-2.004)	0.715		
age				
<50	1			
50-59	0.952 (0.419-2.166)	0.907		
≥60	0.246 (0.204-1.503)	0.246		
Child-Pugh				
A	1			
B	1.208 (0.521-2.802)	0.659		
C	1.110 (0.253-4.863)	0.890		
hepatic encephalopathy				
no	1			
yes	–	–		
ascites				
no	1		1	
yes	2.611 (1.157-5.892)	0.021	1.774 (0.614-5.126)	0.289
TBIL				
<20.5umol/L	1			
≥20.5umol/L	0.811 (0.380-1.729)	0.588		
albumin				
≥35g/L	1			
<35g/L	1.163 (0.523-2.586)	0.710		
Thrombin time				
<18s	1			
≥18s	2.723 (0.808-9.178)	0.106		
HB				
≥110g/L	1			
<110g/L	0.893 (0.380-2.100)	0.795		
PLT				
≥100×109/L	1			
<100×109/L	1.722 (0.815-3.640)	0.154		
ALT				
<50×U/L	1			
≥50×U/L	0.962 (0.454-2.038)	0.920		
HBV DNA				
negative	1		1	
positive	3.451 (1.309-9.102)	0.012	2.597 (0.936-7.207)	0.067
AFP				
<20ug/L	1		1	
≥20ug/L	3.631 (1.449-9.097)	0.006	2.393 (0.638-8.975)	0.196
Tumor stage				
BCLC A	1			
BCLC B	1.283 (0.288-5.706)	0.743		
BCLC C	2.894 (0.628-13.340)	0.173		

## Discussion

The prognosis of advanced HCC is poor, with high recurrence and mortality rate. Early HCC is often treated with radical operations including surgical resection, liver transplantation, and radical TACE (11, 12). At present, domestic patients with recurrent HCC after radical surgery routinely adopt the same treatment as advanced liver cancer, and most of them are systemic drug treatments. The evaluation of their efficacy has not been differentiated from the advanced HCC.

In recent years, the research of molecular targeted drugs in the treatment of HCC has gradually received attention, and multi-kinase inhibitor drugs represented by sorafenib have been widely used in clinical practice. As PD-1/PD-L1 pathway inhibitors were approved by the FDA, immunotherapy targeting immune checkpoints has attracted widespread attention as a new choice for HCC patients. For example, in September 23, 2017, based on the results of the CheckMate 040 trial, the U.S. FDA accelerated the conditional approval of nivolumab as a second-line treatment for HCC, which marked that HCC immunotherapy had advanced to a new stage (13). In addition, the results of the KEYNOTE-224 study showed that compared with placebo, second-line treatment with pembrolizumab can prolong the patient’s OS (13.9 months vs. 10.6 months, HR=0.78, unilateral P=0.023; pre-set α =0.017) and PFS (3.0 months vs. 2.8 months, HR=0.718, unilateral P=0.022; pre-set α=0.002) (14).

Compared with the research of similar drugs abroad, the underlie disease of the research participants in our country is more complicated. For example, the proportion of patients with HBV infection was higher (83.9%), the proportion of subjects with BCLC stage C was as high as 94.9%, the proportion of subjects with extrahepatic metastasis was 81.6%, and patients with AFP≥400ng/mL accounted for 51.2%. The results of this study showed that the median PFS of the TKIs treatment group was 6.2 months, while the median PFS of the TKIs combined with PD-1 treatment group was 14.0 months. The median OS of the TKIs treatment group was 18.0 months, and the median OS of the TKIs combined with PD-1 treatment group was 35.8 months. This is probably attributed to chronic HBV infection, with severe general condition and poor prognosis after recurrence (15).

This study observes and analyzes the efficacy of systemic treatment in patients after radical resection of HCC. Several limitations should be addressed: (1) This is an observational study, in which grouping cannot be randomized. Patients were enrolled from a single center, which made it impossible to avoid some potential confounding biases. (2) The sample size of this study is limited. To some extent, the reliability of the results of the study remained to be improved. (3) The treatment of patients cannot be basically consistent as in clinical drug registration trials. This study is non-interventional, which may lead to bias in the results. This study has preliminarily obtained the curative effect of systemic treatment after radical resection of HCC. Based on the above-mentioned research limitations, it is hoped that follow-up research can overcome the above limitations and obtain more comprehensive research conclusions.

In summary, systemic treatment after liver cancer surgery can positively and effectively improve the survival of patients. Specific populations can choose TKIs combined with PD-1 therapy as the first-line treatment to improve the prognosis of HCC recurrence.

## Data Availability Statement

The original contributions presented in the study are included in the article/**Supplementary Material**. Further inquiries can be directed to the corresponding authors.

## Ethics Statement

The studies involving human participants were reviewed and approved by Medical Ethics Committee of Nanjing Jinling Hospital. The patients/participants provided their written informed consent to participate in this study.

## Author Contributions

XC and YZ conceived of and designed the study. ZL, NH, and XR performed the literature search, generated the figures and tables, and wrote the manuscript. ZL, NH, and XR collected and analyzed the data, and critically reviewed the manuscript. XC and YZ supervised the study and reviewed the manuscript. All authors contributed to the article and approved the submitted version.

## Funding

This study was supported by Natural Science Foundation of Jiangsu Province (BK20200275), and “Pyramid Talent Project”, a three-year action plan for talent development of Second Affiliated Hospital of Naval Medical University.

## Conflict of Interest

The authors declare that the research was conducted in the absence of any commercial or financial relationships that could be construed as a potential conflict of interest.

## Publisher’s Note

All claims expressed in this article are solely those of the authors and do not necessarily represent those of their affiliated organizations, or those of the publisher, the editors and the reviewers. Any product that may be evaluated in this article, or claim that may be made by its manufacturer, is not guaranteed or endorsed by the publisher.
